# Charge Carrier Dynamics in Non-Fullerene Acceptor-Based Organic Solar Cells: Investigating the Influence of Processing Additives Using Transient Absorption Spectroscopy

**DOI:** 10.3390/ma16165712

**Published:** 2023-08-21

**Authors:** Gayoung Ham, Damin Lee, Changwoo Park, Hyojung Cha

**Affiliations:** 1 Department of Energy Convergence and Climate Change, Kyungpook National University, Daegu 41566, Republic of Korea; 2 Department of Hydrogen and Renewable Energy, Kyungpook National University, Daegu 41566, Republic of Korea

**Keywords:** non-fullerene acceptor, charge carrier dynamics, transient absorption spectroscopy, processing additive, organic solar cell

## Abstract

In this study, we present a comprehensive investigation into the charge generation mechanism in bulk-heterojunction organic solar cells employing non-fullerene acceptors (NFAs) both with and without the presence of processing additives. While photovoltaic devices based on Y6 or BTP-eC9 have shown remarkable power conversion efficiencies, the underlying charge generation mechanism in polymer:NFA blends remains poorly understood. To shed light on this, we employ transient absorption (TA) spectroscopy to elucidate the charge transfer pathway within a blend of the donor polymer PM6 and NFAs. Interestingly, the charge carrier lifetimes of neat Y6 and BTP-eC9 are comparable, both reaching up to 20 ns. However, the PM6:BTP-eC9 blend exhibits substantially higher charge carrier generation and a longer carrier lifetime compared to PM6:Y6 blend films, leading to superior performance. By comparing TA data obtained from PM6:Y6 or PM6:BTP-eC9 blend films with and without processing additives, we observe significantly enhanced charge carrier generation and prolonged charge carrier lifetimes in the presence of these additives. These findings underscore the potential of manipulating excited species as a promising avenue for further enhancing the performance of organic solar cells. Moreover, this understanding contributes to the advancement of NFA-based systems and the optimization of charge transfer processes in polymer:NFA blends.

## 1. Introduction

Organic bulk heterojunction (BHJ) solar cells have garnered significant attention due to their ability to produce large-area, flexible, roll-to-roll devices using solution-processed coating technology [1,2,3,4]. The development of non-fullerene acceptors (NFAs) has played a pivotal role in enhancing the power conversion efficiencies (PCEs) of these solar cells. Particularly, the ground-breaking Y6 series of NFAs has led to a substantial increase in the efficiency of single-junction BHJ organic solar cells (OSCs) by over 19% [5,6,7,8]. The achievement of such high-efficiency OSCs necessitates the consideration of various factors, including complementary light absorption between the electron donor and acceptor, appropriate energy level alignment, balanced electron and hole transport, suitable film morphology, and material stability in environmental conditions [9,10,11,12,13,14]. NFAs are particularly well-studied because of their potential to broaden the absorption spectrum into the visible/near-infrared region, reduce voltage losses, and ensure environmental stability compared to conventional fullerene-based acceptors. Consequently, devices utilizing these NFAs have surpassed theoretical predictions, stimulating investigations into the underlying processes that constrain the efficiency of OSCs [15,16,17,18,19,20].

Photocurrent losses constitute a significant factor limiting the PCE of BHJ solar cells. These losses arise from incomplete charge generation and the excitonic nature of organic semiconductors. At room temperature, the low dielectric constant of organic semiconductors causes photogenerated excitons to remain strongly bound as electron–hole pairs. Efficient exciton dissociation necessitates the presence of interfaces between the electron donor (D) and electron acceptor (A) within an interconnected BHJ network. At these interfaces, free charge carriers are generated and subsequently collected at the respective electrodes, resulting in photocurrent generation [21].

The charge carrier lifetime plays a crucial role in determining the diffusion length and the probability of photogenerated charge carriers reaching the electrode in organic solar cells. Particularly, Braun’s modification of Onsager’s original work emphasizes the significance of a finite lifetime for the charge-transfer (CT) state in solids [21]. Striking a balance between optimizing charge separation through the interface surface area and electronic coupling, while maximizing the lifetime of charge-separated states, is essential for efficient charge collection by device electrodes and achieving overall high device efficiencies. However, recombination sites within the bulk heterojunction can reduce the lifetime by creating additional recombination pathways, leading to the loss of excess carriers. Recent research suggests that such recombination sites, often found at intermixed phases, can be influenced through morphology engineering, involving material crystallinity and film processing, which contributes to the improved performance of organic solar cells [5,6,7,8,9]. Therefore, in the thin film state, the slower recombination lifetime is attributed to the dissociation of initially generated charges into separated charges, indicating a transition from intramolecular geminate recombination to bimolecular recombination in the solid film.

The advancement and technological development of the BHJ strategy face inherent limitations. Achieving optimal charge separation and charge transport requires a delicate balance of processing conditions, including the D:A ratio, processing solvents, additives, thermal and/or solvent annealing, and green protocols [22,23]. Notably, the incorporation of processing additives plays a critical role in attaining the desired balance between phase separation and phase purity. Additives usually have a high boiling point and possess selective solubility to one of the active materials. The addition of these additives can usually simultaneously enhance the light absorption and charge carrier mobility as well as suppress charge recombination [24,25,26,27,28,29], which contribute to a higher short-circuit current density (J_SC_) and fill factor (FF), ultimately enhancing the PCE. However, this remains a challenging endeavor, as the current trial-and-error optimization approach does not provide precise control over the vertical component distribution. To date, extensive macroscopic analyses of the physical and chemical properties of OSCs, such as changes in film morphology or charge transport based on molecular structure design, have been conducted. However, to fully comprehend and apply the principles of device operation, a molecular-level understanding of the charge transfer mechanisms is indispensable. By gaining insights into the dynamics of the physical and chemical changes that occur in OSCs during operation and elucidating the microscopic mechanisms underlying these changes, we can establish design principles for materials used in OSCs and enhance their performance. The use of processing additives plays a significant role in achieving these goals.

The PM6:Y6 and PM6:BTP-eC9 blends have emerged as highly promising materials in the field of OSCs, surpassing traditional NFAs. Extensive research has been conducted to investigate the factors contributing to the high efficiency of PM6:Y6 [30], focusing on areas such as efficient charge extraction with lower trap states sites [31], extended exciton lifetimes of up to 1 ns measured by time-correlated single photon counting (TCSPC) analysis [11], barrier-less activation energy elucidated through time-delayed collection field (TDCF) measurements [32], the free charge generation of Y6 [33], S_1_-CT hybridization [34], and more. However, there remains a dearth of studies on the dynamics of free charge carriers with morphology. Consequently, this study aims to compare the behavior of separated charge carriers in Y6 and BTP-eC9 blends, considering the presence or absence of various additives, using nanosecond transient absorption (TA) spectroscopy.

In this article, we introduce TA spectroscopy as a powerful technique for investigating the dynamics of charge transfer and charge separation in OSCs. Based on this technique, we present research results that elucidate the mechanisms of charge separation dynamics and the correlation between molecular structure, properties, and functionality. Furthermore, we explore the role of processing additives in OSCs, which are auxiliary materials incorporated into the formulation of the active layer to improve film morphology and charge transport properties. These additives exert a significant influence on various aspects of device performance, including film formation, crystallinity, and the nanoscale organization of donor and acceptor materials.

## 2. Materials and Methods

### 2.1. Device Fabrication and J–V Characterization

Patterned ITO substrates (Omni Science, South Korea, Rs ≤ 10 Ω/sq) were cleaned by sequential sonication in detergent, water, acetone, and isopropanol for 15 min at each step. Then, the pre-cleaned ITO substrates were treated with oxygen plasma for 20 min. The PEDOT:PSS (Baytron, Germany, Clevious 4083) aqueous solution was filtered (0.45 μm PVDF) and was spin-coated onto the ITO substrates and then annealed in air at 150 °C for 15 min. Organic active materials PM6, Y6, and BTP-eC9 were obtained from Solarmer Materials Inc., China. The active layer was deposited by spin coating from a PM6:NFA (1:1 wt%, 16 mg/mL) solution in the mixed solvent of CF:CN (99.5:0.5 vol%) for PM6:Y6 and CF:DIO (99.5:0.5 vol%) for PM6: BTP-eC9 under 2000 rpm for 60 s on top of PEDOT:PSS in the glove box. Neat films of Y6 and PM6 were also prepared via spin coating using chloroform solutions with concentrations of 8 mg/mL. The film samples were annealed under an N_2_ atmosphere at 100 °C for 5 min. PDINN solution (1 mg/mL in methanol) was spin-coated onto the active layer at 4000 rpm for 30 s. Then, the devices were immediately put into the chamber for electrode deposition. Finally, the device fabrication was completed by thermal evaporation of 100 nm Ag as the electrode. The effective device area was defined to be 0.045 cm^2^ controlled with a shadow mask. *J*–*V* characteristics were measured using a Xenon lamp at AM1.5 solar illumination with a Sol3A class AAA solar simulator (Newport, RI, USA, model 94023A, 2 × 2 in.) calibrated to a silicon reference cell with a Keithley 2400 source meter, correcting for the spectral mismatch.

### 2.2. UV-Vis Absorption and Photoluminescence Spectroscopy

UV-Visible spectra of thin films on glass substrates were measured with a Cary 60 UV-Vis spectrophotometer (Agilent, Santa Clara, CA, USA). The photoluminescence (PL) spectra were measured with an FS5 spectrofluorometer (Edinburgh Instrument, Livingston, UK). All film samples were spin-cast on glass substrates.

### 2.3. Transient Absorption Spectroscopy

Nanosecond (ns) TA data of thin films on glass substrate are obtained using a pump and probe nanosecond transient spectroscopy system. The system comprises a transient absorption spectrometer and a regenerative amplified Nd: YAG laser (EL-YAG) with a pulse width of 6–8 ns, which is capable of generating both visible pulses (532 nm) and UV pulses (355 nm) through a third harmonic generator. The probe beam is derived from a 150 W Xenon lamp, which is reflected off the powder sample and then passes through a monochromator before reaching a PMT-980 photodiode detector. To simultaneously capture data on two different time scales, the comprehensive L900 spectrometer software (V9.4.3) package is utilized, and the ns-s signal is sampled using an oscilloscope (Tektronix MDO3022, Beaverton, OR, USA). Excitation fluences are measured using a pyroelectric energy sensor.

## 3. Results and Discussion

**Figure 1:**
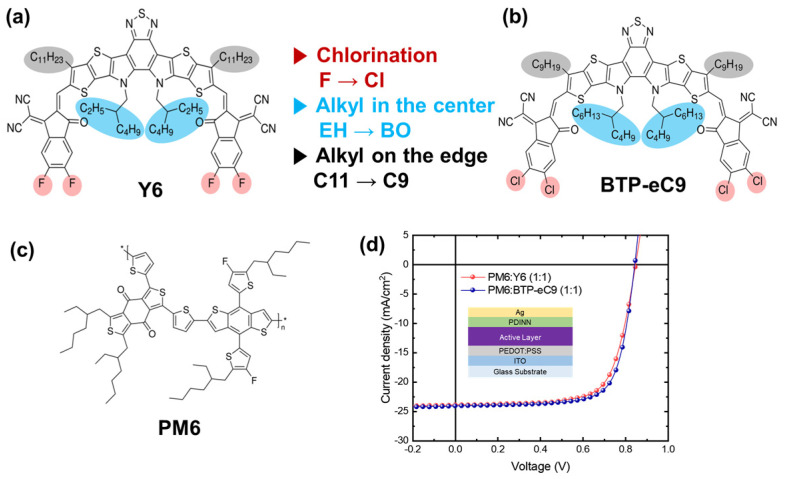
The molecular structures of (**a**) Y6 and (**b**) BTP-eC9 as electron acceptors, and (**c**) PM6 as an electron donor; (**d**) *J*–*V* characteristics of PM6:Y6 and PM6:BTP-eC9 solar cells (inset: a device architecture).

The blend employed in this study comprises the polymer donor PM6 and the small molecule acceptors Y6 or BTP-eC9, as depicted in Figure 1a–c. PM6 is a fluorinated medium bandgap polymer based on the benzodithiophene-alt-benzo [1,2-b:4,5-c′]dithiophene-4,8-dione backbone, while Y6 is a non-fullerene acceptor (NFA) consisting of an electron-deficient centrally fused conjugated ring (dithienthiophen [3.2-b]-pyrrolobenzothiadiazole) flanked by 2-(5,6-difluoro-3-oxo-2,3-dihydro-1H-inden-1-ylidene)malononitrile end-capping units. On the other hand, BTP-eC9 differs from Y6 in terms of electronegativity and side chain optimization, as depicted in Figure 1a,b. Polymer:NFA blend solar cells can be produced using an inverted structure, utilizing ZnO as an electron transport layer (ETL) and MoO_3_ as a hole transport layer (HTL), which can offer greater air stability compared to conventional configurations. However, for PM6:Y6 blends, conventional devices incorporating PEDOT:PSS as an HTL and PDINN as an ETL exhibit significantly higher photovoltaic performance than inverted device structures [30]. In this work, conventional organic solar cells utilizing the PM6:Y6 or PM6:BTP-eC9 blends yield PCEs around 15% with processing additives, as shown in Figure 1d and Table S1. The processing additives can effectively modify the aggregation behavior of materials, resulting in well-defined bi-continuous interpenetrating networks. The phase separation also leads to a stabilized energy state within pure domains, which serves as a driving force for charge separation. The photocurrent generation depends on the excitonic behavior before charge separation. As a result, the kinetics of the charge-transfer (CT) state afterward play a crucial role in the overall process of photocurrent generation. For optical experiments, film samples were prepared via spin coating on a glass substrate.

**Figure 2:**
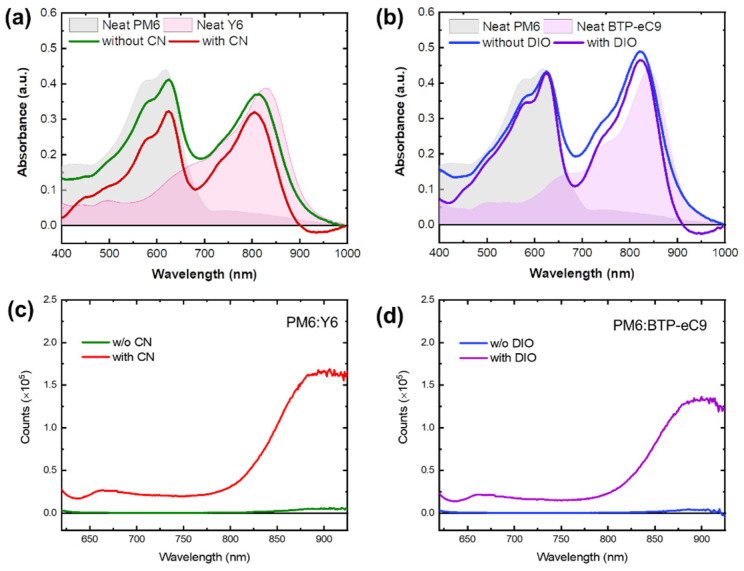
UV-Vis absorption spectra of (**a**) PM6:Y6 and (**b**) PM6:BTP-eC9 blend films with and without additives including PM6 and Y6 neat films. Photoluminescence spectra of (**c**) PM6:Y6 and (**d**) PM6:BTP-eC9 blend films with and without additives excited at 600 nm, respectively.

The absorption spectra of neat films and donor–acceptor (D:A) blend films were measured and are presented in Figure 2a,b. The optical absorption spectra of PM6 and Y6 demonstrate complementary absorption in the visible to near-infrared range. The neat PM6 film exhibits a wide range of photon harvesting, which is characterized by a prominent absorption peak at 630 nm. In addition, the PM6 blend film displays a red-shift of 6 nm, indicating a less crystalline film morphology, resembling that of the neat film. On the other hand, the Y6 and BTP-eC9 films exhibit strong photon-harvesting abilities in the longer wavelength range with absorption peaks observed at 820 nm and 850 nm, respectively, clearly illustrating complementary absorption spectra with PM6. However, the NFA in the blend exhibits a blue shift of 20 nm compared to the neat films, suggesting the intercalation of NFA molecules into the PM6 phase, thereby impeding intermolecular stacking. Interestingly, the addition of processing additives does not significantly affect the absorbance in both PM6:Y6 an PM6:BTP-eC9 blends.

Steady-state photoluminescence (PL) was carried out to obtain insight into the exciton dissociation efficiency of the blends in the presence of a processing additive, as shown in Figure 2c,d. After adding the processing additive to the blend of the donor and acceptor, an obvious increase in PL intensity was observed for both blends compared to the blends without any additives. The increased PL intensity with processing additives would suggest that the use of processing additives could lead to better phase separation of the active layer.

**Figure 3:**
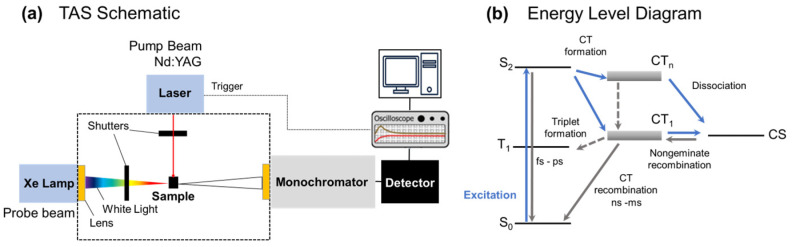
(**a**) Schematic representation of the setup for nanosecond transient absorption spectroscopy and (**b**) general conceptual model for understanding charge carrier dynamics in organic solar cells. Energy level diagram illustrating the formation of multiple electronic CT states following CT from excited state S_1_. All CT states either dissociate to charge-separated (CS) states or decay to the ground state S_0_ (CT recombination). Separated charges may non-geminately recombine to the CT state. The presence of low-energy triplet states at the donor or acceptor enables triplet (T_1_) back energy transfer from the CT state.

To investigate charge generation and recombination in organic active layers, ns-TA spectroscopy is employed as a relatively straightforward pump-probe technique, enabling the observation of excessive absorption of photogenerated charge carriers. This technique requires high detection sensitivity and accurate measurement of the signal size at low excitation densities to avoid nonlinear and saturation effects. Excitation is achieved using picosecond laser pulses, while the Si photodiode detects the excessive absorption signal after a delay time in the nanosecond range. The magnitude of transient polaron absorption (ΔOD, change in optical density) directly indicates the quantum efficiency of charge generation. A sufficiently low excitation density is utilized to minimize non-geminate recombination within the measured time range. This straightforward approach facilitates comparative studies of charge generation efficiency across different photoactive layer materials, blend compositions, and processing conditions (refer to Figure 3).

In organic semiconductor films, excitons lifetimes exhibit shorter than 1 nanosecond and diffusion distances shorter than 20 nm. These excitons undergo charge transfer at the interface between the donor and acceptor. Subsequently, excitons can either dissociate into free charges or persist as bound electron–hole pairs at the donor/acceptor interface. The CT state, representing the electron–hole pair at the interface, is readily identified by the red-shifted absorption and emission compared to the pure material. Both geminate a recombination of Coulombically bound CT states, and dissociation into free charges has been observed at these interfaces.

**Figure 4:**
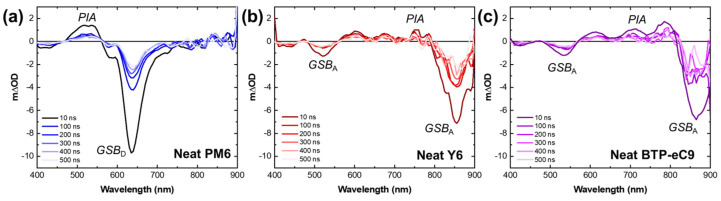
Transient absorption spectra of neat films of (**a**) PM6; (**b**) Y6; (**c**) BTP-eC9 probed at the visible region, 400–900 nm.

Figure 4 presents the TA spectra obtained from pristine PM6, Y6, and BTP-eC9 samples upon excitation at 355 nm. Upon optical pumping, PM6 exhibits primary excitation characterized by a bleach band centered at 630 nm (GSB_D_) and a photoinduced absorption (PIA) band centered at 520 nm. At a delay time of 500 ns, long-lived species are observed at 630 nm. Similarly, the excitation of Y6 and BTP-eC9 displays two bleach bands centered at 520 nm and 850 nm (GSB_A_) along with a PIA band spanning the range of 600 to 800 nm. Once again, at a delay time of 500 ns, the presence of long-lived species is observed.

**Figure 5:**
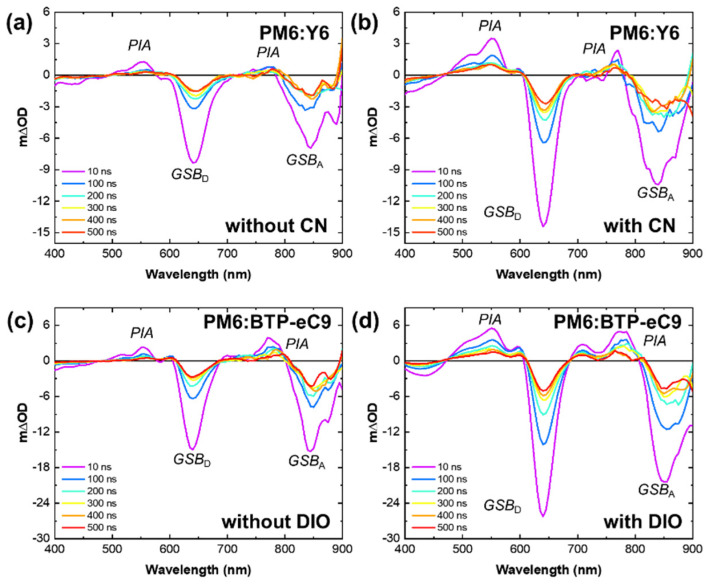
Transient absorption spectra of (**a**,**b**) PM6:Y6 and (**c**,**d**) PM6:BTP-eC9 films with and without additives probed at the visible region, 400–900 nm.

Figure 5 depicts the TA spectra obtained from PM6:Y6 and PM6:BTP-eC9 films, both with and without additives, probed in the visible region upon excitation at 355 nm. Based on the TA data for the pristine materials, the primary excitation in PM6:NFA blends is observed through two bleach bands centered at 630 nm (GSB_D_) and 850 nm (GSB_A_). In the presence of processing additives, the overall TA signal is significantly enhanced, indicating a stronger phase separation and reduced CT state recombination through intermediate phases.

**Figure 6:**
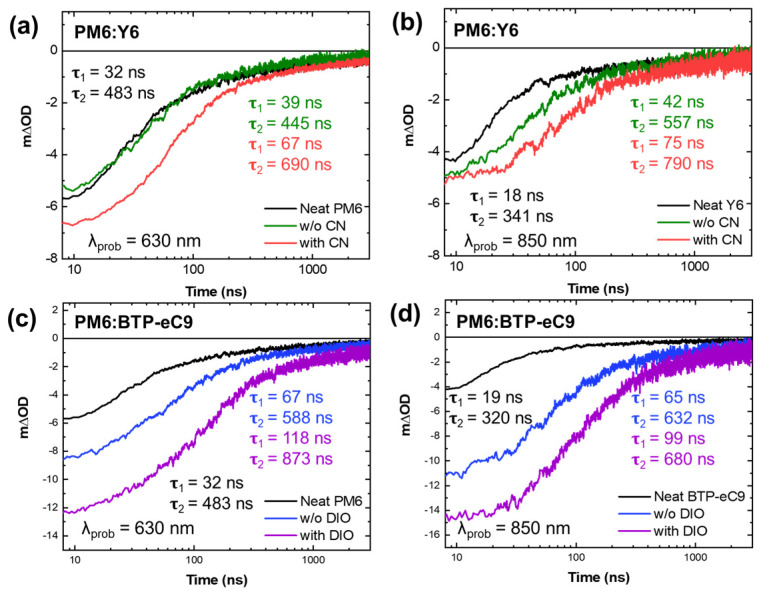
Transient absorption spectra of (**a**,**b**) PM6:Y6 and (**c**,**d**) PM6:BTP-eC9 films including neat films with and without additives, probed at 650 nm for (**a**,**c**) and at 850 nm for (**b**,**d**), respectively.

The presence of additives in blend films contributes to the formation of a purer phase, consequently leading to an increase in the radiative recombination of excitons, as shown in PL spectra. On the other hand, this phase separation appears to make slow non-radiative charge recombination. In Figure 6, we present a comparison of the charge carrier kinetics at 630 nm (GSB_D_) and 850 nm (GSB_A_). It is observed that the carrier lifetime in the blend films is prolonged with the incorporation of additives, indicating that the carriers are not solely excitons (carrier lifetime calculated by a fitting equation in supporting information). Furthermore, incorporating additional processing additives leads to an approximately twofold increase in the carrier lifetime for both GSB signals. When comparing the PM6:Y6 and PM6:BTP-eC9 blends, the BTP-eC9 blends exhibit higher TA intensity and a longer carrier lifetime, which can be attributed to their higher crystallinity. This suggests an efficient charge separation process from CT states to free charges, thereby significantly influencing the device performance.

**Figure 7:**
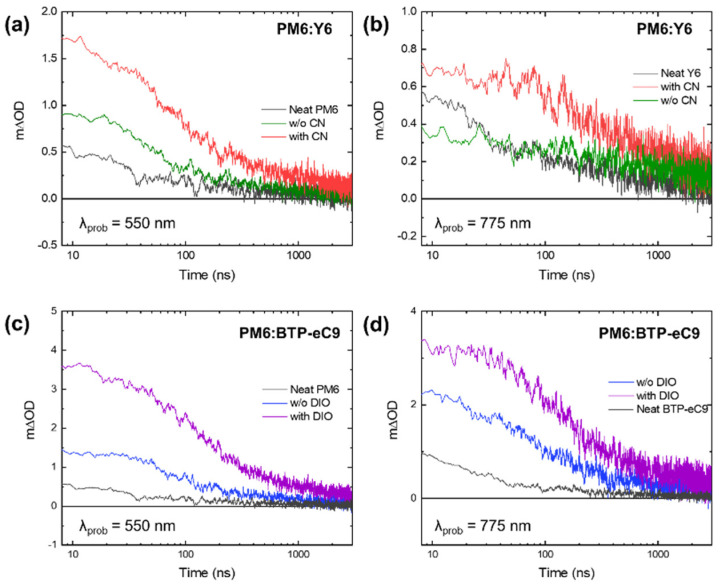
Transient absorption spectra of (**a**,**b**) PM6:Y6 and (**c**,**d**) PM6:BTP-eC9 films including neat films with and without additives probed at 550 nm for (**a**,**c**) and at 775 nm for (**b**,**d**), respectively.

To comprehensively investigate charge carrier dynamics in excited states, we conducted an analysis of photo-induced absorption (PIA) features at 550 nm and 775 nm for both PM6:Y6 and PM6:BTP-eC9 blends, both with and without processing additives as illustrated in Figure 7. Notably, the TA spectra exhibiting a long-lived excited state of the PIA feature at 775 nm in this study are in good agreement with the previous literature [35]. This feature is likely attributed to positive polarons resulting from hole transfer from the acceptor NFA to the polymer donor PM6. Conversely, the PIA feature at 550 nm is indicative of negative polarons generated through electron transfer from PM6 to NFA. Both PIA features are linked with the bimolecular recombination of free polarons within the charge separation state. Furthermore, they exhibit prolonged carrier lifetimes when processing additives are presented, as indicated in Table S2. This enhancement is possibly connected to efficient charge separation accompanied by well-defined phase separation. Moreover, these results are consistent with the carrier dynamics observed in GSB features. Additionally, in comparison to the PM6:Y6 blend, the PM6:BTP-eC9 blend exhibited a more intense signal at 8 ns and a prolonged carrier lifetime, resulting in a higher photocurrent due to stronger intermolecular interaction.

## 4. Conclusions

In this study, the PM6:Y6 and PM6:BTP-eC9 blends have demonstrated significant potential in the field of OSCs. These blends, consisting of the polymer donor PM6 and small molecule acceptors Y6 or BTP-eC9, have exhibited impressive PCEs of approximately 16% or higher. The optical absorption spectra reveal complementary absorption characteristics between the polymer donor and small molecule acceptors, contributing to efficient light harvesting. Through nanosecond TA measurements, we gain valuable insights into the charge carrier dynamics within the blends. The incorporation of processing additives in the blends plays a crucial role in enhancing phase separation and reducing CT state recombination, as evidenced by the observed increase in overall transient absorption signals. Furthermore, the presence of additives leads to extended carrier lifetimes in the blend films, indicating the existence of charge carriers beyond excitons. Notably, the introduction of additional processing additives further extends the carrier lifetime, resulting in improved charge separation efficiency. Comparing the PM6:Y6 and PM6:BTP-eC9 blends, the BTP-eC9 blends exhibit higher transient absorption intensity and longer carrier lifetime, which is attributed to their higher crystallinity. This underscores the importance of charge separation dynamics and morphology in achieving efficient OSC performance. These findings emphasize the significance of investigating charge separation mechanisms and optimizing the morphology of OSCs. Further research in this area is vital to drive the continued progress and advancement of OSC technology.

## Data Availability

Not applicable.

## Supplementary Materials

The following supporting information can be downloaded at: https://www.mdpi.com/article/10.3390/ma16165712/s1. Table S1: Device performance of PM6:Y6 and PM6:BTP-eC9 with processing additives; Table S2: Transient absorption kinetics of PM6:Y6 and PM6:BTP-eC9 blend films including neat films with and without additives probed at 550 nm and at 775 nm, respectively.

## References

[1] Yu G., Gao J., Hummelen J.C., Wudl F., Heeger A.J. (1995). Polymer Photovoltaic Cells: Enhanced Efficiencies via a Network of Internal Donor-Acceptor Heterojunctions. Science.

[2] Granström M., Petritsch K., Arias A.C., Lux A., Andersson M.R., Friend R.H. (1998). Laminated fabrication of polymeric photovoltaic diodes. Nature.

[3] Günes S., Neugebauer H., Sariciftci N.S. (2007). Conjugated Polymer-Based Organic Solar Cells. Chem. Rev..

[4] Blom P.W.M., Mihailetchi V.D., Koster L.J.A., Markov D.E. (2007). Device Physics of Polymer: Fullerene Bulk Heterojunction Solar Cells. Adv. Mater..

[5] Zhu L., Zhang M., Xu J., Li C., Yan J., Zhou G., Zhong W., Hao T., Song J., Xue X. (2022). Single-junction organic solar cells with over 19% efficiency enabled by a refined double-fibril network morphology. Nat. Mater..

[6] Zheng Z., Wang J., Bi P., Ren J., Wang Y., Yang Y., Liu X., Zhang S., Hou J. (2021). Tandem Organic Solar Cell with 20.2% Efficiency. Joule.

[7] Han C., Wang J., Zhang S., Chen L., Bi F., Wang J., Yang C., Wang P., Li Y., Bao X. (2023). Over 19% Efficiency Organic Solar Cells by Regulating Multidimensional Intermolecular Interactions. Adv. Mater..

[8] Wang J., Zheng Z., Zu Y., Wang Y., Liu X., Zhang S., Zhang M., Hou J. (2021). A Tandem Organic Photovoltaic Cell with 19.6% Efficiency Enabled by Light Distribution Control. Adv. Mater..

[9] Peet J., Kim J.Y., Coates N.E., Ma W.L., Moses D., Heeger A.J., Bazan G.C. (2007). Efficiency enhancement in low-bandgap polymer solar cells by processing with alkane dithiols. Nat. Mater..

[10] Karuthedath S., Gorenflot J., Firdaus Y., Chaturvedi N., De Castro C.S.P., Harrison G.T., Khan J.I., Markina A., Balawi A.H., Pena T.A.D. (2021). Intrinsic efficiency limits in low-bandgap non-fullerene acceptor organic solar cells. Nat. Mater..

[11] Classen A., Chochos C.L., Lüer L., Gregoriou V.G., Wortmann J., Osvet A., Forberich K., McCulloch I., Heumüller T., Brabec C.J. (2020). The Role of Exciton Lifetime for Charge Generation in Organic Solar Cells at Negligible Energy-Level Offsets. Nat. Energy.

[12] Wei Q., Yuan J., Yi Y., Zhang C., Zou Y. (2021). Y6 and its derivatives: Molecular design and physical mechanism. Natl. Sci. Rev..

[13] Natsuda S.-I., Sakamoto Y., Takeyama T., Shirouchi R., Saito T., Tamai Y., Ohkita H. (2021). Singlet and Triplet Excited-State Dynamics of a Nonfullerene Electron Acceptor Y6. J. Phys. Chem. C.

[14] Natsuda S., Saito T., Shirouchi R., Sakamoto Y., Takeyama T., Tamai Y., Ohkita H. (2022). Cascaded energy landscape as a key driver for slow yet efficient charge separation with small energy offset in organic solar cells. Energy Environ. Sci..

[15] Armin A., Li W., Sandberg O.J., Xiao Z., Ding L., Nelson J., Neher D., Vandewal K., Shoaee S., Wang T.A. (2021). History and Perspective of Non-Fullerene Electron Acceptors for Organic Solar Cells. Adv. Energy Mater..

[16] Li C., Zhou J., Song J., Xu J., Zhang H., Zhang X., Guo J., Zhu L., Wei D., Han G. (2021). Non-Fullerene Acceptors with Branched Side Chains and Improved Molecular Packing to Exceed 18% Efficiency in Organic Solar Cells. Nat. Energy.

[17] Li G., Feng L.-W., Mukherjee S., Jones L.O., Jacobberger R.M., Huang W., Young R.M., Pankow R.M., Zhu W., Lu N. (2022). Non-Fullerene Acceptors with Direct and Indirect Hexa-Fluorination Afford > 17% Efficiency in Polymer Solar Cells. Energy Environ. Sci..

[18] Wang Y., Xue J., Zhong H., Everett C.R., Jiang X., Reus M.A., Chumakov A., Roth S.V., Adedeji M.A., Jili N. (2023). Control of the Crystallization and Phase Separation Kinetics in Sequential Blade-Coated Organic Solar Cells by Optimizing the Upper Layer Processing Solvent. Adv. Energy Mater..

[19] Yuan J., Zhang Y., Zhou L., Zhang G., Yip H.-L., Lau T.-K., Lu X., Zhu C., Peng H., Johnson P.A. (2019). Single-Junction Organic Solar Cell with over 15% Efficiency Using Fused-Ring Acceptor with Electron-Deficient Core. Joule.

[20] Gasparini N., Jiao X., Heumueller T., Baran D., Matt G.J., Fladischer S., Spiecker E., Ade H., Brabec C.J., Ameri T. (2016). Designing ternary blend bulk heterojunction solar cells with reduced carrier recombination and a fill factor of 77%. Nat. Energy.

[21] Clark T.M., Durrant J.R. (2010). Charge Photogeneration in Organic Solar Cells. Chem. Rev..

[22] Taheri-Ledari R., Ganjali F., Zarei-Shokat S., Saeidirad M., Ansari F., Forouzandeh-Malati M., Hassanzadeh-Afruzi F., Hashemi S.M., Maleki A. (2022). A Review of Metal-Free Organic Halide Perovskite: Future Directions for the Next Generation of Solar Cells. Energy Fuels.

[23] Pezhooli N., Rahimi J., Hasti J., Maleki A. (2022). Synthesis and evaluation of composite TiO2@ZnO quantum dots on hybrid nanostructure perovskite solar cell. Sci. Rep..

[24] Lee K., Ma W.L., Brabec C.J., Moon J.S., Kim J.Y., Lee K., Bazan G.C., Heeger A.J. (2008). Processing Additives for Improved Efficiency from Bulk Heterojunction Solar Cells. J. Am. Chem. Soc..

[25] Lou S.J., Szarko J.M., Xu T., Yu L., Marks T.J., Chen L.X. (2011). Effects of Additives on the Morphology of Solution Phase Aggregates Formed by Active Layer Components of High-Efficiency Organic Solar Cells. J. Am. Chem. Soc..

[26] Gu Y., Wang C., Russell T.P. (2012). Multi-Length-Scale Morphologies in PCPDTBT/PCBM Bulk-Heterojunction Solar Cells. Adv. Energy Mater..

[27] Sun Y., Welch G.C., Leong W.L., Takacs C.J., Bazan G.C., Heeger A.J. (2012). Solution-processed small-molecule solar cells with 6.7% efficiency. Nat. Mater..

[28] Lee T.K., Park S.Y., Walker B., Ko S.-J., Heo J., Woo H.Y., Choi H., Kim J.Y. (2017). A universal processing additive for high-performance polymer solar cells. RSC Adv..

[29] Guo X., Cui C., Zhang M., Huo L., Huang Y., Hou J., Li Y. (2012). High efficiency polymer solar cells based on poly(3-hexylthiophene)/indene-C70 bisadduct with solvent additive. Energy Environ. Sci..

[30] Wen Z.-C., Yin H., Hao X.-T. (2021). Recent progress of PM6:Y6-based high efficiency organic solar cells. Surf. Interfaces.

[31] Wu J., Lee J., Chin Y.-C., Yao H., Cha H., Luke J., Hou J., Kim J.-S., Durrant J.R. (2020). Exceptionally Low Charge Trapping Enables Highly Efficient Organic Bulk Heterojunction Solar Cells. Energy Environ. Sci..

[32] Perdigón-Toro L., Zhang H., Markina A., Yuan J., Hosseini S.M., Wolff C.M., Zuo G., Stolterfoht M., Zou Y., Gao F. (2020). Barrierless Free Charge Generation in the High-Performance PM6:Y6 Bulk Heterojunction Non-Fullerene Solar Cell. Adv. Mater..

[33] Fu Y., Lee T.H., Chin Y.-C., Pacalaj R.A., Labanti C., Park S.Y., Dong Y., Cho H.W., Kim J.Y., Minami D. (2023). Molecular orientation-dependent energetic shifts in solution-processed non-fullerene acceptors and their impact on organic photovoltaic performance. Nat. Commun..

[34] Eisner F., Azzouzi M., Fei Z., Hou X., Anthopoulos T.D., Dennis T.J.S., Heeney M.J., Nelson J. (2019). Hybridization of Local Exciton and Charge-Transfer States Reduces Non-Radiative Voltage Losses in Organic Solar Cells. J. Am. Chem. Soc..

[35] Wang R., Zhang C., Li Q., Zhang Z., Wang X., Xiao M. (2020). Charge Separation from an Intra-Moiety Intermediate State in the High-Performance PM6:Y6 Organic Photovoltaic Blend. J. Am. Chem. Soc..

