# Supplementary material for: Charge Carrier Dynamics in Non-Fullerene Acceptor-Based Organic Solar Cells: Investigating the Influence of Processing Additives Using Transient Absorption Spectroscopy
Source: Materials (Basel). 2023 Aug 21;16(16):5712. doi: 10.3390/ma16165712 (PMC10456882; doi:10.3390/ma16165712)
Supplement: Supplementary file 1 [file materials-16-05712-s001.zip › materials-2561926-supplementary.pdf]

Supporting Information

Table S1. Device performance of PM6:Y6 and PM6:BTP-eC9 with processing additives.

Photoactive layer	J_{sc} (mA/cm^{-2})	V_{oc} (V)	*FF* (%)	*PCE* (%)
PM6 : Y6	23.980	0.849	70.52	14.36
PM6:BTP-eC9	24.16	0.845	73.25	14.96

Table S2. Transient absorption kinetics of PM6:Y6 and PM6:BTP-eC9 blend films including neat films with and without additives probed at 550 nm and at 775 nm, respectively.

**Probe wavelength**	**Samples**	**PM6:Y6**				**PM6:BTP-eC9**			
		A_1	τ_1 (ns)	A_2	τ_2 (ns)	A_1	τ_1 (ns)	A_2	τ_2 (ns)
550 nm	neat PM6	0.70	28	0.23	642	0.70	28	0.23	642
	w/o additive	0.82	46	0.23	603	0.77	74	0.22	762
	w/ additive	0.72	79	0.20	1036	0.73	123	0.21	829
775 nm	neat NFA	0.61	27	0.25	545	0.74	21	0.20	429
	w/o additive	0.25	141	0.33	1543	0.73	88	0.16	765
	w/ additive	0.43	154	0.37	1169	0.74	140	0.21	1725

Fitting Equation for calculating carrier lifetime:

$$I(t) = A_1 \exp\left(-\frac{t-t_0}{\tau_1}\right) + A_2 \exp\left(-\frac{t-t_0}{\tau_2}\right) \qquad (1)$$

where, the start time of the decay process is represented by t_0, while τ_1 and τ_2 denote the first and second order decay times, respectively. A_1 and A_2 are weighting coefficients assigned to each decay channel.
